# Species Delimitation in a Polyploid Group of Iberian *Jasione* (Campanulaceae) Unveils Coherence between Cryptic Speciation and Biogeographical Regionalization

**DOI:** 10.3390/plants12244176

**Published:** 2023-12-15

**Authors:** Miguel Serrano, Santiago Ortiz

**Affiliations:** Department of Botany, Faculty of Pharmacy, University of Santiago de Compostela, 15782 Santiago de Compostela, Spain; santiago.ortiz@usc.es

**Keywords:** biogeography, cryptic species, Mediterranean flora, speciation, taxonomy

## Abstract

Groups with morphological stasis are an interesting framework to address putative cryptic species that may be hidden behind traditional taxonomic treatments, particularly when distribution ranges suggest disjunct and environmentally heterogeneous biogeographic patterns. New hypotheses of delimitation of evolutionary independent units can lead to the identification of different biogeographic processes, laying the foundation to investigate their historical and ecological significance. *Jasione* is a plant genus with a distribution centered in the Mediterranean basin, characterized by significant morphological stasis. Within the western Mediterranean *J*. gr. *crispa* species complex, *J. sessiliflora* s.l. and allied taxa form a distinct group, occupying environmentally diverse regions. At least two ploidy levels, diploid and tetraploid, are known to occur in the group. The internal variability is assessed with phylogenetic tools, viz. GMYC and ASAP, for species delimitation. The results are compared with other lines of evidence, including morphology and cytology. The fitting of distribution patterns of the inferred entities to chorological subprovinces is also used as a biogeographical and environmental framework to test the species hypothesis. Despite the scarcity of diagnostic morphological characters in the group, phylogenetic delimitation supports the description of at least one cryptic species, a narrow endemic in the NE Iberian Peninsula. Moreover, the results support the segregation of a thermophilic group of populations in eastern Iberia from *J. sessiliflora*. Ploidy variation from a wide geographical survey supports the systematic rearrangement suggested by species delimitation. Taxonomic reorganization in *J. sessiliflora* s.l. would allow ecological interpretations of distribution patterns in great accordance with biogeographical regionalization at the subprovince level, supporting geobotanical boundaries as a framework to interpret species ecological coherence of cryptic lineages. These results suggest that species differentiation, together with geographic isolation and polyploidization, is associated with adaptation to different environments, shifting from more to less thermophilic conditions. Thus, the recognition of concealed evolutionary entities is essential to correctly interpret biogeographical patterns in regions with a complex geologic and evolutionary history, such as the Mediterranean basin, and biogeographical units emerge as biologically sound frameworks to test the species hypothesis.

## 1. Introduction

The accelerated global biodiversity decay [1] urges the need to identify units of conservation representing evolutionary significant entities, the most important of which is the species [2]. Species have been defined as independent evolving groups of organisms that are genetically and phenotypically distinct from other groups, a definition that encapsulates both the pattern of species and the causes of the pattern [3]. Independent evolution leads to divergence, due to the interruption of cohesive processes related to gene flow. Species would be the most resolved unit of diversity with independent evolution. Although populations could develop in isolation, they still are not identified as independently evolving entities on account of insufficient time to accumulate diagnosable signatures [3]. The accumulation of genetic differences and actual independent evolutionary paths are not always correlated with diagnosable differences in morphological traits, as happens in taxonomic groups with a high degree of morphological stasis and which, therefore, are prone to cryptic speciation [4,5]. Cryptic species would remain undetected, which hampers ecological approaches studying these complexes. The reason is that cryptic entities would have evolved without clear morphological differences. However, accumulation of differences in environmental preferences is probable due to the evolutionary paradigm of niche conservatism between sister species [6,7]. Researchers studying groups with hidden entities could inadvertently mix information that should be treated separately, which could lead to meaningless biological hypotheses. Finally, and more importantly, these species are excluded from legal lists and, therefore, conservation policies will be blind to them [8]. Evolutionary lineages with morphological stasis can be composed by species with few, if any, clearly discriminating morphological characters, although genetic differentiation and interruption of gene flow between the different lineages could have a relatively long evolutionary history. However, other phenotypic traits can be investigated to detect significant differences among them those related with ecological preferences or habitats [9,10]. The use of molecular information can uncover hidden significant specific divergence supported by species delimitation methods. It allows researchers to focus on the detection of other corroborating signatures of speciation, and on the knowledge of the expected distribution of discontinuities between populations. The evolutionary significant unit (ESU) concept [11] defines species as units with reciprocal monophyly in at least one molecular marker plus at least other defining trait, including morphology, distribution, or, in general, reciprocal monophyly in another characteristic [10]. Although the original ESU concept includes differences in distribution among the defining traits, a direct application of reciprocal monophyly and separated ranges would lead to an inflation of species recognition in many taxa. Nevertheless, it could be advisable to include information from species distribution if it indirectly offers relevant information about the environmental envelope of the putative evolutionary entity. The environmental niche in which a species thrives is ultimately conditioned by intrinsic physiological constraints. Evolved ecological tolerances make up the fundamental niche, that is, the potential environmental space that a species could occupy [12]. However, the realized niche is the one that can be inferred from distribution data, and is also conditioned by biotic interactions and the historical ability of the species to overcome barriers to dispersal [12]. This is illustrated by cases where an invasive species with clear niche shifts between the native and recently invaded range [13]. Thus, distribution ranges are a translation to the geographical space of the evolved optimal environmental preferences of a species that allow it to thrive and successfully interact with other species, conditioned by random opportunities to dispersal. Biogeographical regionalization makes a good hypothetical framework to test hypotheses of evolutionary independent lines (i.e., ESUs or species), as it integrates not only information from the environmental preferences of the geographically encompassed organisms in each sector, but about the occurring communities, and, therefore, their internal between-species interactions. In this aspect, the adequacy of a species or a lineage to certain biogeographical sectors, but not to others, could provide more information about its singularity than the mere environmental information obtained from geographical occurrences of the species. Nevertheless, there are different approaches to biogeographic regionalization, such as those which aim to establish homologous biotic units based on sympatric distributions of endemic species (i.e., areas of endemism) together with complementary criteria, although the existence of different concretizations remains a problem [14,15,16]. Scientists from the geobotanic school have proposed a hierarchized sectorization of biogeographic units widely used in phytosociological research, based on the distribution of species and vegetal communities, in addition to information from other fields, mainly bioclimatology, geology, soil science and geography, establishing typological units to classify the different territories of the Earth [17]. The hierarchical typologies of organization are normally shared among the different schools in their higher categories, such as kingdom, region, province, and district, including intermediate categories, like subregions or subprovinces [16]. Lower levels of organization are more controversial, and inferior categories of the geobotanic school (e.g., sector, tesela, etc.) have not gained support for biogeographical regionalization [16]. Therefore, the subprovince level of regionalization can be both a consensual typology and at the same time divide the territory into a sufficient number of units to identify patterns in closely related species, in case of the absence of strict niche conservatism.

The task of assigning populations to cryptic species must be based on observed patterns. One species’ property is that species constitute ecological units [3], which makes the species the main unit for ecological and biogeographical models of diversity. Although there exist ecotype variations and adaptive polymorphism within species [18,19] the criterion of ecological coherence has a special importance in species definitions, particularly in organisms with scarce morphological distinctiveness [3]. The present work is based on the conceptual framework that ecological coherence within species and ecological differentiation between species could produce diagnosable biogeographic patterns, and if this is the case, the identified patterns should support cryptic entities inferred from other lines of evidence, namely from the DNA sequence methods. Nevertheless, the accumulation of lines of evidence is needed, as the blind use of molecular methods can lead to incorrect inferences. The results of empirical methods for species delimitation based on nucleotidic information, regardless of the use of either single-locus sequences or genome-scale data, can be misled by some evolutionary processes specific to the group under study. Processes derived from hybridization events and introgression, and recent radiations and incomplete lineage sorting, can blur the inference [20,21]. Thus, complex phylogeographic histories and semipermeable species boundaries [21] make the accumulation of diverse lines of evidence important, particularly in close evolutionary entities within the same ploidy level. It is especially important at the beginning of the speciation process when different independent lines of evidence offer frequent conflicting signals. It is fundamental to identify those evolutionary independent entities in the group under study not only to unravel the evolutionary events behind the process, but to apply conservation polices when necessary due to the accelerated process of global biodiversity loss. The use of sequence-based species delimitation (SD) methods could be considered the first exploratory step to set the species hypothesis in an integrative taxonomy process that should be coupled with expert knowledge in the group of interest. The type of sequence information for systematic studies currently ranges from nuclear or organelle genes to genomic approaches, including genome-wide SNP data, plastomes, or complete genomes. The use and potential of these sources of information has been discussed in several works (e.g., [22,23,24,25]). While genomic data clearly increase analytic resolution, the pattern that emerges is that Sanger sequencing and genomic approaches generally agree in their results and that no one technology from sequence information gives a final answer in species delimitation studies [26]. Information from genomics does not alter the need for operational criteria, particularly in hybrids or polyploids, and emphasis has been placed on incorporating data on ecology, physiology, or reproductive biology to address species delimitation [25]. Thus, the empirically suggested species are ready to be supported or discarded when faced with other lines of evidence, such as testing if the proposed species fit or behave in a differentiated manner.

In this work, the model group was a complex of taxa and diverged populations of the genus *Jasione*, namely *Jasione sessiliflora* and morphologically related taxa, all belonging to the wider *J. crispa* polyploid group. The genus *Jasione* is characterized by a marked morphological stasis that led to conflicting taxonomic treatments (e.g., [27,28]). The *J. sessiliflora* group of study is formed by Iberian endemic taxa, with at least two ploidy levels within, diploid and tetraploid, and whose populations have been assigned different taxonomic names. The more firmly established taxa of the group in current taxonomic treatments are *Jasione sessiliflora*, considered a tetraploid distributed in the Central and northern half of the Iberian Peninsula, and *Jasione crispa* subsp. *tomentosa*, with its allied taxon *J. crispa* subsp. *mariana* [29]. These latter taxa have distribution ranges in the Central Iberian Peninsula, although in more southernly areas than *J. sessiliflora* [30]. Both are considered diploids, although Sales and Hedge [31] included in the variability of *J. crispa* subsp. *mariana* tetraploid populations that have been described under the name *J. crispa* subsp. *segurensis*. Finally, two disjunct Mediterranean groups of populations have been assigned to *J. sessiflora*, namely *J. sessiliflora* subsp. *appressifolia* in Castellón and the Valencia mountains and a population in the Prades mountains, in Tarragona, which was originally described as *J. crispa* var. *praelittoralis*, a treatment abandoned since the description [29,31]. Although different groups of populations are assigned to different names, the taxonomic resolution has been considered complicated and requires detailed studies, as the diagnostic characters are frequently absent [29,31]. Therefore, it makes an excellent model group on which to use species delimitation tools and on which to use the delimited entities as a specific hypothesis to test by identifying discontinuous patterns.

In summary, the aim of this study is to detect species in the cryptic diploid/tetraploid group of *J. sessiliflora* and allied taxa by using two complementary methods of molecular species delineation, ASAP and GMYC, whose results are considered hypotheses of species identification. To validate the species hypotheses, different complementary lines of evidence are studied, including ploidy variation and morphological characters. Finally, a main objective of this work is to appraise whether the suggested species represented by molecular lineages are in accordance with biogeographical subprovinces in a way that clear biogeographical patterns can be identified as an expression of differently evolved realized niches. Under this premise, biogeographical subprovinces would represent an ecologically sound framework to test species as environmentally coherent unique evolutionary trajectories.

## 2. Results

### 2.1. Species Delimitation with ASAP

**Figure 1:**
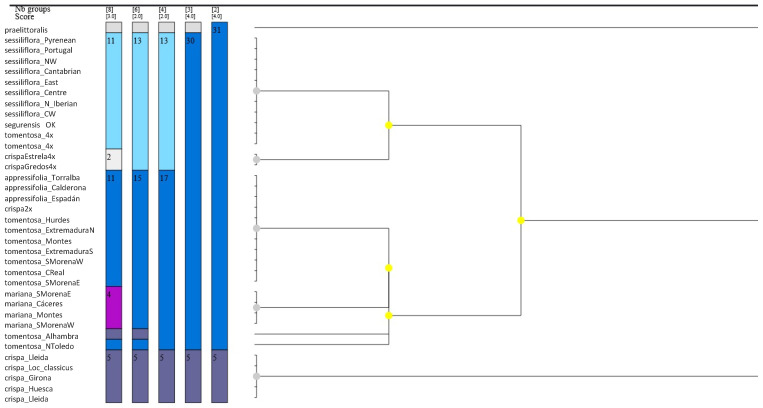
ASAP species delimitation tree calculated with the Kimura-2-parameter model. Some terminals within a group from different localities share identical haplotypes. The third column is recovered as the most likely species delimitation hypothesis, suggesting four species. Nodes of this tree are color-coded depending on their *p*-value (the darker the more the node differs from a panmictic species). The colorful fields indicate the groups of species. Each field contains the number of individuals. Above bars it is indicated the asap-score (the lower value) and number of species (the upper value) recognized for the dataset.

ASAP generated five possible partitions, but only the two best asap-scores according to [32] were considered for discussion, as in [33]. The first and second best scored partition schemes divided the dataset into four and six species, respectively. Both partitions had similar values, with identical asap-scores in the preferred Kimura two-parameter (K80) model in the analysis with unrepeated sequences (Table 1) and slightly better probability of panmixia (*p*-value) for the four species partition (0.71 vs. 0.73, averaged probability values of the three substitution models in the four and six species partitions, respectively, the lower ranked the better). ASAP ranked the four species partition scheme first because of its better probability. Moreover, it overpasses the reference threshold distance (0.0005). The distance inferred by ASAP between panmictic populations and diverged species was 0.00061 for the four species partition and 0.000454 for the six species partition (Table 1). Similar results were obtained in the analysis from all geographic areas that included repeated haplotypes (Table 2), with somewhat better panmixia probability for the four species scheme (0.53 vs. 0.65, averaged probability values of the three substitution models in the four and six species partitions, respectively, the lower ranked the better). Both analyses identified that the same four groups as distinct species in the best-ranked partition were (1) *Jasione crispa* var. *praelittoralis*, (2) *Jasione crispa* subsp. *crispa*, (3) all tetraploid representatives (*Jasione sessiliflora*, *Jasione crispa* subsp. *segurensis*, and tetraploid populations of *Jasione crispa* from Serra da Estrela and *Jasione* “*tomentosa*” 4x), and (4) all remaining diploid taxa (*Jasione crispa* subsp. *tomentosa*, *Jasione crispa* subsp. *mariana*, *Jasione sessiliflora* subsp. *appressifolia*, and other unassigned diploid populations). The six-partition schemes were similar but they separated two diploid populations of *Jasione tomentosa* with somewhat divergent sequences. (Figure 1). Other biologically meaningful schemes, like a partition with three species, also identified *J. crispa* subsp. *crispa* and *J. crispa* subsp. *praelittoralis* as independent species, lumping the remaining populations in a group; however, this was with a considerably worse asap-score of 5. Given the comparable results between both partitions the four species partition is preferred, since it has better probability values and higher threshold distances, considering that the six species partitions had an over-split resulting from a phenetic rather than phylogenetic method [32].

ASAP delimitation was defined by evaluating both the partitions with the first and second best asap-scores, according to [32].

### 2.2. Species Delimitation with GMYC

The species delimitation scheme estimated by the GMYC single-threshold method in the dataset with sequences collapsed to unique haplotypes varied from two to four species (Table 3). The topologies inferred from a constant clock rate prior led to four maximum likelihood entities (*praelittoralis*, *crispa* Pyrenean, tetraploid haplotypes, remaining diploid haplotypes), while in the genealogy inferred from a relaxed clock rate, the GMYC method estimated two maximum likelihood entities (*crispa* Pyrenean, all other haplotypes). Maximum likelihood confidence intervals were narrower when the expected branching pattern was based on a constant coalescent model than when using a Yule model. The LRT results were not significant in the three cases, although they were closer to significant values for the topology inferred with a coalescent prior. Conversely, the GMYC support (AIC values) for the basal diverging node was highest in the Yule model.

**Figure 2:**
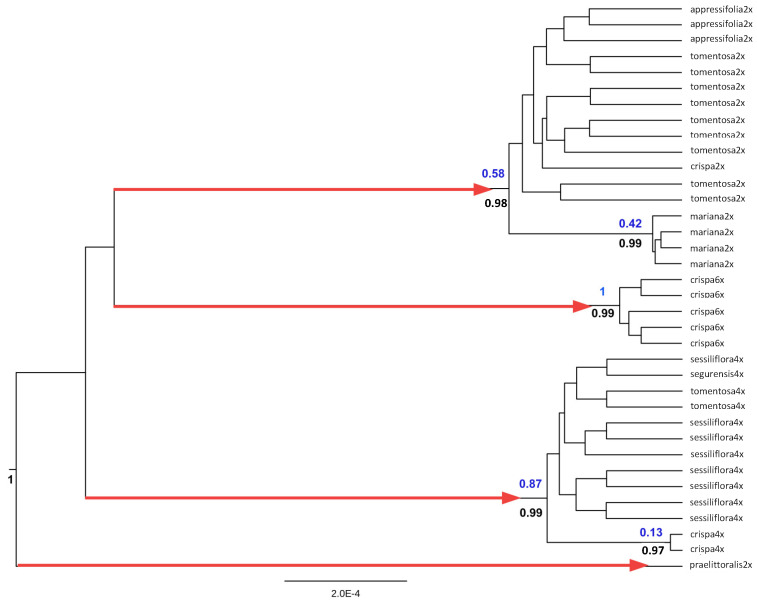
GMYC species delimitation in a Bayesian genealogy inferred from coalescent and constant clock rate priors. The four lineages suggested as putative species are indicated by red arrows. Posterior probabilities of well-supported nodes in the Bayesian phylogeny are indicated in black under branches. GMYC support (AIC values) of the same nodes are indicated in blue above branches.

**Figure 3:**
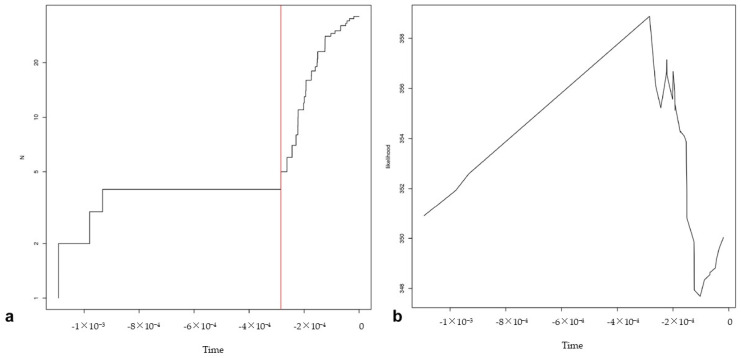
GMYC species delimitation from the “populations” dataset analysis. (**a**) Number of lineages through time (as accumulated mutations) and inferred threshold for species delimitation. The red line express the boundary between the parts of the phylogeny in which the branching shows a behavior following a population (coalescent) model (to the right of the line) and those parts that follow a speciation (Yule) model (to the left of the line). (**b**) Profile of the estimated likelihood through evolutionary time.

Nevertheless, given the narrower confidence intervals and better likelihood ratios of the analyses on the topology from the coalescent branching and constant clock rate priors, the current authors used a topology inferred with these prior parameters for the populations dataset (with repeated sequences). The resulting maximum likelihood estimates recovered 4 species (*praelittoralis*, *crispa*, tetraploid haplotypes, remaining diploid haplotypes), with the narrowest confidence intervals (4–9 species), comparatively good GMYC support, and highly significant LRT results (Table 3). Therefore, the species scheme is congruent with two of the three analyses carried out with the “haplotypes” dataset, but well supported by likelihood ratios test. The nodes leading to the four lineages estimated as putative species had high posterior probability support in the Bayesian inference, but not all clades with high posterior probability support are recovered by GMYC as putative species (Figure 2). The profile of the likelihood and the number of lineages through time, expressed as accumulated mutations, since the genealogy is not time-calibrated, with the inferred threshold between divergence (speciation) and coalescent (populational) events, are shown in Figure 3.

### 2.3. Other Lines of Evidence: Ploidy Variation and Morphology

**Figure 4:**
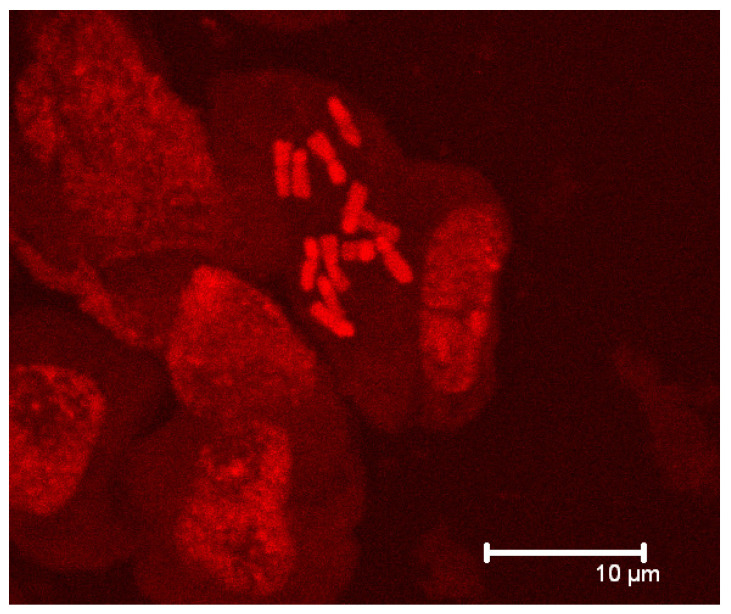
Diploid metaphase (2n = 12) of *Jasione crispa* var. *praelittoralis* from Prades, Tarragona.

The cytological analyses revealed that *Jasione crispa* var. *praelittoralis* is a diploid taxon with 2n = 12 chromosomes (Figure 4). *Jasione sessiliflora* var. *appressifolia* is also diploid, with 2n = 12 chromosomes in the studied populations. *Jasione crispa* subsp. *tomentosa* showed variation in ploidy level among populations, with most of its populations being diploid (2n = 12) and four tetraploid populations (2n = 24). *Jasione sessiliflora* was tetraploid (2n = 24). Six populations from the Central System with conflicting taxonomic assignation, either considered *Jasione sessiliflora* or *Jasione crispa* subsp. *tomentosa*, were identified as diploids (2n = 12). These populations show morphological differences with typical *Jasione sessiliflora* and are considered “unassigned diploids” in this work hereinafter. The two investigated populations of *Jasione crispa* subsp. *segurensis* were tetraploids (2n = 24), as expected. A few populations assigned to *Jasione crispa* from the western part of the Central Iberian System range unexpectedly turned out to be tetraploids (2n = 24), which is consistent with its phylogenetic position among the tetraploids as *J. sessiliflora* subsp. *sessiliflora*.

**Figure 5:**
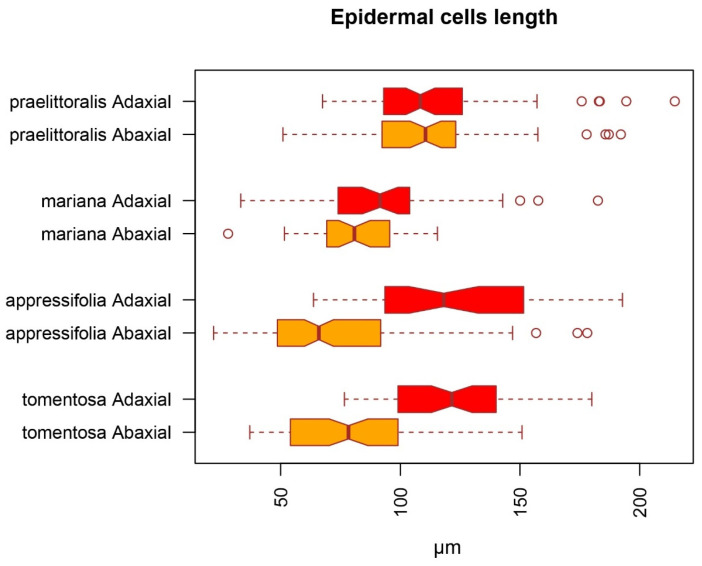
Length of the maximum dimension of adaxial epidermal cells (in red) and abaxial epidermal cells (in orange) in the leaves in diploid groups identified by ASAP and GYMC. Relative differences in size support the identity of the “*appressifolia* group” (diploid populations of *J. crispa* subsp. *tomentosa* and *J. sessiliflora* subsp. *appressifolia*) and differentiation from *J. crispa* var. *praelittoralis* and *J.crispa* subsp. *mariana*.

Measurements of morphological traits allowed the identification of informative variation between the taxa coherent with the delimitation analyses. The study of the relative sizes of adaxial and abaxial epidermal cells, considered by [34] as one of the few anatomical traits of taxonomical significance in Iberian *Jasione* species, allowed the identification of morphological differences even among the phenotypically quite homogenous diploid lineages of *Jasione crispa* var. *praelittoralis*, *Jasione crispa* subsp. *tomentosa*, and *Jasione sessiliflora* subs. *appressifolia*. The results are coherent with the specific groups suggested by ASAP and GYMC, namely between the diploid populations of *Jasione crispa* subsp. *tomentosa* and *Jasione sessiliflora* subs. *appressifolia* on one side, and the putative cryptic species represented by *Jasione crispa* var. *praelittoralis* on the other (Figure 5). Bokhari and Sales [34] considered that *Jasione crispa* subsp. *tomentosa* was anatomically characterized among other members of its group by adaxial epidermal leaf cells much larger that abaxial ones. Thus, these results, together with their shared ploidy level and cpDNA polymorphisms, contribute to support the close proximity of *Jasione crispa* var. *praelittoralis* and *Jasione crispa* subsp. *tomentosa*, suggesting they form a single evolutionary group of geographically disjunct populations. For the sake of simplicity, they will be addressed as *appressifolia* group hereinafter. *Jasione crispa* subsp. *mariana*, representing a morphologically more differentiated group that frequently occurs in sympatry with *Jasione crispa* subsp. *tomentosa*, maintains its differentiation from the latter in this subtle trait.

**Figure 6:**
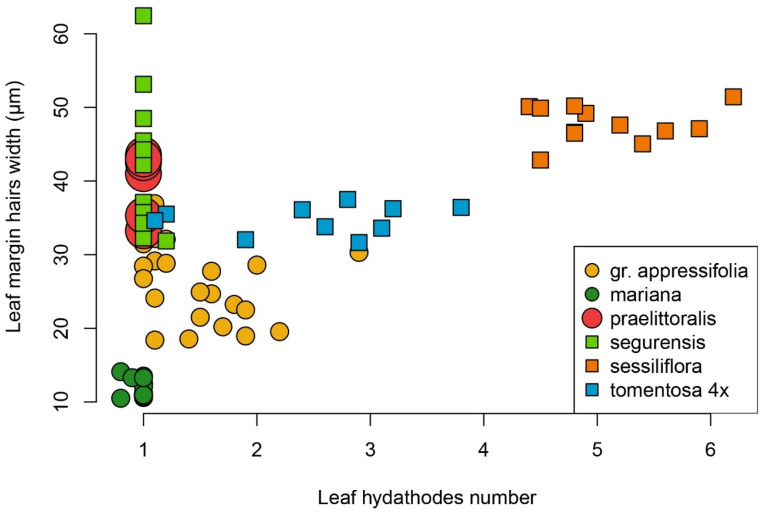
Leaf margin hair width (in μm) vs. number of hydathodic structures, with values representing the average of each character in individuals of *J. crispa* var. *praelittoralis*, *J. crispa* subsp. *mariana*, *J. crispa* subsp. *segurensis*, tetraploid populations of *J. crispa* subsp. *tomentosa*, *J. sessiliflora* subsp. *sessiliflora*, and “gr. *appressifolia*” (including diploid populations of *J. crispa* subsp. *tomentosa* and *J. sessiliflora* subsp. *appressifolia*).

Hydathodic traits and trichome robustness are also consistent with the species delimitation from phylogenetic tools. Although the distribution of variability within each taxon is not completely unimodal in both traits, the dispersion of values is illustrative enough to reveal interesting discontinuities (Figure 6). Some correlation exists between ploidy level and hair width, with higher values occurring in tetraploid individuals. *J. sessiliflora* subsp. *appressifolia* and *J. crispa* susbp. *tomentosa* are represented together in Figure 6 as “gr. *appressifolia*” to reduce figure complexity and given their complete overlap in the investigated traits. The name “*tomentosa* 4x” is reserved to those populations with intermediate characteristics between *J. sessiliflora* and *J. crispa* subsp. *tomentosa* identified as tetraploids. *Jasione crispa* subsp. *praelittoralis* tends to have larger hair widths despite being diploid, but still shows some overlap with some individuals, with only one hydathodic structure per leaf, as in the “*appressifolia*” group.

### 2.4. Consistency between Biogeographical Regionalization and Distributions of the Inferred Evolutionary Significant Units

**Figure 7:**
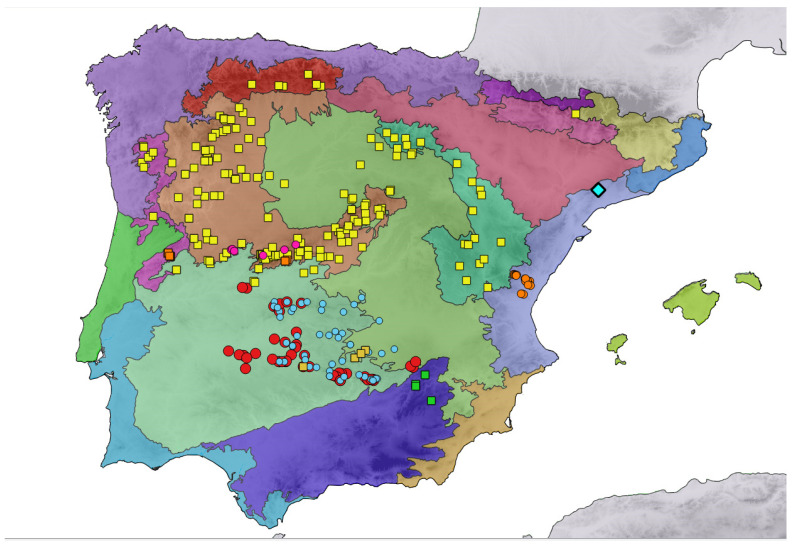
Biogeographical subprovinces of the Iberian Peninsula (image modified from [17], where the colour codes of subprovinces are shown) and populations of the different groups investigated of *Jasione*. Diploids: *J. crispa* var. *praelittoralis* (blue diamond), *J. crispa* var. *appressifolia* (orange circles), *J. crispa* var. *tomentosa* (blue circles), and unassigned *J. crispa* diploids from the Central System (pink circles). Tetraploids: *J. sessiliflora* subsp. *sessiliflora* (light yellow squares), *J. crispa* subsp. *tomentosa* 4× (dark yellow squares), and *J. crispa* 4× from the Central System (orange squares). The map shows taxonomical groups and within-group variability in ploidy, whether these groups are suggested as species by ASAP and GMYC analyses or not.

The results show a high degree of concordance between the geographic distributions of the investigated groups and the biogeographical regionalization of geobotanic subprovinces of the Iberian Peninsula [17]. In Figure 7, group differentiation is maximized according to existing taxonomic categories and ploidy levels identified in this work. The tetraploid *J. sessiliflora* subsp. *sessiliflora* has the widest range, occurring in five subprovinces, mostly in the inner northern half of the Iberian Peninsula where the climate shows subcontinental and sub-Mediterranean features [35]. In the eastern Iberian Peninsula, *J. sessiliflora* subsp. *sessiliflora* populations are close to populations of the diploid *J. sessiliflora* subsp. *appressifolia*, albeit both lineages respect the biogeographical boundaries of the Oroiberian subprovince and the coastal Valencian subprovince, respectively. There is a general pattern of diploids keeping their ranges within warm subprovinces, either in the in east for *J. crispa* var *praelittoralis* and *J. crispa* var. *appressifolia* or in the southwest, for *J. crispa* subsp. *tomentosa* and *J. crispa* subsp. *mariana*. A few diploid populations morphologically and genetically akin to the “*appressifolia* group” occur in mountain ranges around the boundary between the southwestern Lusitania and Extremadura, and the northwestern Carpetania and León subprovinces. The remaining tetraploid populations have a narrow distribution and are represented by *J. crispa* subsp. *segurensis*, endemic to the Betica subprovince, tetraploid populations so far assigned to *J. crispa* subsp. *tomentosa*, mostly in the Castillian subprovince, and tetraploid populations of *J. crispa* from the western Central System range. ASAP and GYMC analyses nest these groups within the molecular variability of *J. sessiliflora* subsp. *sessiliflora*.

**Figure 8:**
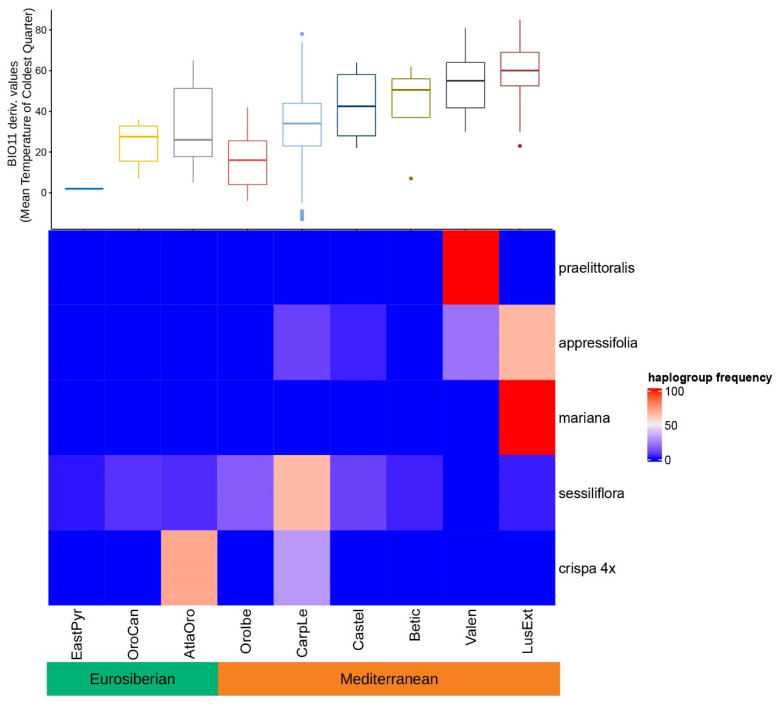
Heatmap of haplotype frequencies of the four haplogroups with posterior probability support and GMYC support (indicated on the right) in the biogeographic subprovinces of the Iberian Peninsula (indicated at the bottom) [17]. The scatter plot of the mean temperature values of the coldest quarter of the year represents the values obtained from populations of the model group in each subprovince.

**Figure 9:**
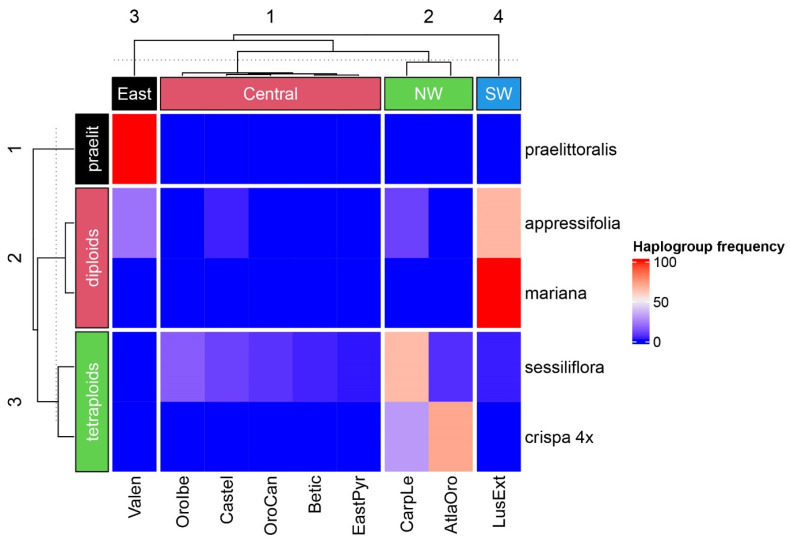
Double K-means split and clustering heatmap analysis based on frequencies of each haplogroup with Bayesian posterior probability and GMYC supports (indicated on the right) in the biogeographic subprovinces of the Iberian Peninsula (indicated at the bottom). The dashed line cuts at the group level. Geographical groups are represented at the top. Species delimitation suggested by biogeographic clustering is based on the occurrence of the haplogroups in the different subprovinces, and is represented by the groups “*praelittoralis*”, “diploids”, and “tetraploids” (indicated on the left).

The heatmap analyses made on the haplogroups with GMYC support show a clear biogeographic structure (Figure 8 and Figure 9). Diploid groups, represented by the clusters of the “*praelittoralis*”, “*mariana*”, and “*appressifolia*” haplogroups, are concentrated in the Lusitania and Extremadura and Valencia subprovinces, and in all cases are within the Mediterranean region. These are the most thermic of all subprovinces with occurrences of representatives of the model group (Figure 8). The tetraploids represented by the “*sessiliflora*” and “*crispa* 4×” haplogroups show a preference for colder subprovinces, penetrating into the Eurosiberian region in the Orocantabrian and the Atlantic Orolusitania subprovinces, although the latter has transitional features between the two major climatic regions [36,37]. The k-means clustering analysis based on haplotype frequencies established a clear hierarchy coincident with the GMYC and ASAP methods (Figure 9). Thus, haplogroups are clustered in three main groups based on their biogeographic features (i.e., clustered following their relative frequency across the sectorized biogeographic space) supporting the hypothesis of three species suggested by the species delimitation phylogenetic tools. The first is exclusive for *J. crispa* var. *praelittoralis* with the haplogroup “*praelittoralis*”, the second encompasses the remaining diploids represented by haplogroups “*appressifolia*” and “*mariana*”, and the third encompasses the tetraploids and is represented by haplogroups “*sessiliflora*” and “*crispa* 4×”. The analysis also split and clustered the biogeographic subprovinces based on the haplotype frequencies, identifying four geographical groups of subprovinces in the Iberian Peninsula (North-West, Central, South-West and East) relevant for the variability in the investigated plastid DNA variability in the studied model group.

## 3. Discussion

Species delimitation is a complex task in recently diverged lineages and in those that underwent radiation but did not fit in models of adaptive innovations reflected in morphological phenotype variation [38]. Evolutionary morphological stasis would result in cryptic speciation, making it necessary to address species delimitation [39], although the results could vary regarding the lines of evidence used [10]. The greater the number of concordant lines of evidence, the more reliable the results. In this work, two species delimitation phylogenetic methods were used (ASAP and GMYC), which agreed, suggesting the recognition of at least three species within the diploid–tetraploid cryptic complex of *J. sessilifilora*. These results were complemented by several concordant lines of evidence including cytology and morphology, so assigning populations to species could be confidently based on observable patterns of relative discontinuity, although these were not immediately evident.

The ploidy level of diverse taxa is published for the first time in this work, with the ploidy distribution also investigated at the population level across the Iberian Peninsula. Thus, *Jasione sessiliflora* subsp. *appressifolia* and *Jasione crispa* var. *praelittoralis* are identified as diploids, which supports the ASAP and GMYC species delimitation hypotheses separating the former from the tetraploid *Jasione sessiliflora* subsp. *sessiliflora* and the latter from the hexaploid *Jasione crispa* subsp. *crispa*. In summary, the three main lineages are identified as species. One group encompasses the tetraploid populations of *J. sessiliflora* subsp. *sessiliflora*, a few tetraploid populations of the western Central System range previously assigned to *J. crispa* subsp. *crispa* [31], tetraploid populations with intermediate characters between *J. sessiliflora* and *J. crispa* subsp. *tomentosa*, and the narrow endemic *J. crispa* subsp. *segurensis*. The second group includes most diploid populations, with *J. crispa* subsp. *mariana*, *J. crispa* subsp. *tomentosa*, and *J. sessiliflora* subsp. *appressifolia*. The two latter taxa actually form a single cryptic unity. Finally, the third lineage exclusively includes the narrow endemic *J. crispa* var. *praelittoralis*, revealing it to be an unexpected cryptic diploid species.

The results of the multi-evidence approach supported the inferred three groups, but also suggest that internal variability within the first and second groups requires taxonomic reorganization and the need for nomenclatural updating in agreement with the identified evolutionary trajectories, as they firmly refute previous taxonomical proposals. As an example, the results contradict the inclusion proposed by Ref. [29] of *J. crispa* subsp. *segurensis* within the variability of *J. crispa* subsp. *tomentosa*. Similarly, the proposal of Ref. [40] of including the populations of the coastal mountains of Valencia and Castellón (Eastern Spain) in *J. sessiliflora* (as *J. sessiliflora* subsp. *appressifolia*) is not supported either. Reciprocal monophyly is a property of those groups of populations that become different evolutionary units, although the differentiation degree could be not important enough to designate species, particularly when secondary contact allows pervasive introgression processes. Species delimitation tools would refuse to recognize not strongly differentiated lineages as species even if they could represent interesting evolutionary units. This pattern was identified for *J. crispa* subsp. *mariana* within the diploid group, and for the tetraploid populations of *J. crispa* from the Central System. Both groups formed statistically well supported subclades in the Bayesian phylogenetic analysis used to generate a tree for GMYC (Figure 2). However, while none of them were recognized as different species by ASAP or GMYC, their evolutionary significance probably makes these lineages worthy of more detailed studies. In particular, the maintenance of important phenological and morphological specificities, probably due to phenological assortative mating, in *J. crispa* subsp. *mariana* despite general sympatry with *J. crispa* subsp. *tomentosa*, could justify a particular taxonomic rank. Regarding the latter, all investigated lines of evidence support a close affinity with *J. sessiliflora* subsp. *appressifolia*, representing two disjunct groups of populations of the same lineage. Therefore, a number of new nomenclatural combinations should be addressed, with the most urgent task being to elevate *Jasione crispa* var. *praelittoralis* to the species rank. This plant is a very narrow endemic with a small number of individuals (L. Sáez, com. pers.) occurring in the Prades mountains in Southern Catalonia. Possibly very endangered, this taxon represents a relict diploid lineage that arose after the first lineage bifurcation in the group, and it deserves immediate conservation measures.

Ecological uniqueness is one of the properties that characterize species [3]. But, to what extent could the fitting of a species to the boundaries of biogeographical regionalization explain its ecological uniqueness? Our results suggest that environmental information synthesized by geobotanic regionalization has great explanatory power to individualize species, at least in the investigated group. Species delimitation and biogeographic coherence appear to be mutually explicative. Differentiated suitability to contiguous or proximate biogeographic units of the delimited species was tested in this work as a complementary line of evidence to support evolutionary differentiation in neighboring lineages. Ultimately, we aimed to test whether the delimited species showed internal coherence in realized niches by using this as a proxy of their adequacy to biogeographical regionalization. The results consistently supported this theoretical conceptualization. The distribution of frequencies in the subprovinces of five haplogroups was assessed by non-aprioristic k-means split analysis. The results fulfilled the best expectations of species ecological distinctiveness with differentiated realized niches identified by proxy from biogeographical coherence. Haplogroups were biogeographically organized in full agreement with the delimited species proposed by the ASAP and GMYC methods. Biogeographical coherence would probably be result of adaptive processes to different niches to reduce the cohesive effects of gene flow. As a polyploid system is involved in this case, complementary explanations of niche shift to scape minority cytotype exclusion should be considered [41,42], as has been discussed in other polyploid systems of the genus *Jasione* [43,44]. Although reticulate patterns of evolution are typically present in polyploid groups, which might hamper evolutionary interpretation [45,46], cpDNA variation has not shown any relevant signature of hybrid processes in the studied model group. Altogether, this points to a niche shift across the lineage from ancestral thermophilic environments that characterize the diploid groups to colder ecological conditions in the polyploids. Niche conservatism, or evolutionary preservation of ancestral environmental requirements [7,47], seems to maintain most of the diploid populations within the boundaries of the warm subprovinces of the Southwest and the Mediterranean East. Tetraploid populations show wider geographic dispersion, but the general trend is for them to occur in colder areas. Although all populations of the studied group occur in mid- (or, more rarely, high-) elevation mountain ranges, which confers sub-Mediterranean climatic conditions, the inferred species grouping the tetraploid populations concentrates in areas within the subprovinces with colder thermotypes [36]. Climatic variability within subprovinces is expressed in terms of bioclimatic belts of temperature (i.e., thermotypes) and precipitation (i.e., ombrotypes) [36]. In this regard, the diploids in the Lusitanian and Extremadura and Valencia subprovinces appear confined to meso-Mediterranean and subhumid belts. Meanwhile, the areas inhabited by the tetraploids are characterized by higher average elevation and lower mean temperatures, with predominant supra-Mediterranean thermotypes in the Carpetania and León and the Oroiberian subprovinces. In the same way, there is a clear increase in precipitation, as those areas frequently show humid (or even hyper-humid) ombrotypic features. Despite its southern geographical locations, this climatic signature (humid and supra-Mediterranean belts) is also detected in the mountain areas of the Bética subprovince occupied by the tetraploid “*sessiliflora*” haplogroup, represented by populations of *J. crispa* subsp. *segurensis*. Overall, a shift correlated with increase in ploidy from warmer and drier to colder and wetter conditions seems plausible. However, as far as water requirements are concerned, the shift may only be apparent. The subprovinces with diploid presence (Lusitania and Extremadura and Valencia) are characterized by high environmental humidity of maritime origin, with frequent advection topographic fogs at the mountain ranges where the diploids occur. This frequently overlooked phenomenon probably compensates the water regimes of these areas, which allows the presence in these mid-elevation mountain ranges of sub-Mediterranean plant species with mesic requirements [35,48].

Temperature seems to be a more determining factor in understanding the change that occurred in the study group. This pattern of warm-to-cold niche evolution across a lineage has been found in other European plant groups [49,50] and is consistent with the hypothesis of climatic refugia during the Pleistocene for more conservative lineages and the opportunity to adapt to available colder environments for more evolutionary dynamic lineages [51]. Although the geobotanical regionalization has come under criticism and is not universally followed [16], our results support the use of biogeographical regionalization as an ecologically sound framework to interpret the evolutionary significance of plant lineages. It could allow a window into the ecological cohesion of lineages which are potentially cryptic species. These represent unnoticed unique evolutionary trajectories [4], and ignorance of their existence is in itself a threat factor, which should encourage scientists to carry out special research and conservation efforts in this area.

## 4. Materials and Methods

### 4.1. Plant Material

We explored all localities from which specimens have been described for the treated names, encompassing seven taxa. Then, populations covering the whole distribution ranges of the members of the group were sampled (41 populations for molecular markers, 83 populations for ploidy assessment). Most of the material was sampled in the field, ranging from three to five individuals from each population, although some populations are represented by single individuals or by herbarium specimens. Populations from all the biogeographical subprovinces [17] with representatives of the group have been included in the study, with a special effort to avoid sampling bias. In addition to representatives of the group of interest, the species delimitation analyses included individuals from populations of the nominal subspecies, such as the hexaploid *Jasione crispa* subsp. *crispa*, including samples from the type locality (locus classicus) at Eina Mountain [29].

### 4.2. DNA Sequences and Phylogenetic Analysis

Total genomic DNA was extracted using either the modified cetyltrimethylammonium bromide (CTAB) method [52] or a Nucleo Spin Plant II (Macherey-Nagel) commercial kit. In both cases, 0.6 uL of 2-Mercaptoethanol was added during the first liquid step. Foliar tissue was homogenized either with a manual grinder or using a Mikro dismembrator (Sartorius). DNA concentration and quality were assessed with a Nanodrop 2000C (Thermo Scientific, Waltham, MA, USA). Four plastid DNA regions were amplified, the *trn*H-*psb*A intergenic espacer, the trnL-trnF intergenic spacer, the 3′ end of the intron of the *ndh*F gene, and the *trn*T(UGU)-*trn*Q intergenic spacer. Primers were obtained from the bibliography or designed here. Initially, it was intended to amplify the *rps*16-*trn*Q intergenic spacer with primers from Ref. [53], but poor amplification led us to design internal primers specifically for *Jasione*. After alignment and comparison with plastome sequences of members of the Campanulaceae in the GenBank, it turned out that the conserved regions where the designed primers are anchored belong at the end of *the trnL*(UUA)-*trnT*(UGU) spacer and the *trnT*(UGU) gene and the *trnQ* gene region. This can be explained by the massive rearrangements experienced by plastid genomes in *Jasione* and, in general, by the Campanulaceae family [54]. Therefore, the amplified marker will be denominated *trnT*(UGU)-*trnQ*. Primer sequences and their bibliographic references are listed in Table 4. PCR conditions were 1 cycle of 95 °C/3 min, 34 cycles of 94 °C/30 s; 54 °C /30 s; 72 °C/2 min, and a final extension cycle of 72 °C/10 min. PCR products were firstly assessed on 1.5% agarose electrophoresis gels stained with the nucleic acid staining solution RedSafe (iNtRON biotechnology, Seoul, Republic of Korea) and purified with an E.S.N.A. Cycle Pure Kit. (Omega). DNA Sanger sequencing was carried out in an 8-capillary 3500 analyzer (Applied Biosystems, Foster City, CA, USA) by the DNA sequencing service of the University of Santiago de Compostela, Spain, or Software Sequencing Analysis 5.2 (Applied Biosystems) by STAB VIDA, Portugal. GenBank accession numbers are listed in Table S1.

### 4.3. Ploidy Level Assessment

Direct chromosome counting was carried out in metaphases from root apices. Root tips measuring c. 5 mm were excised from cultivated plants grown from field collected individuals or from germinated seeds. All plants and seedlings were acclimated to outdoor conditions and were directly moved from temperatures around 15 °C or lower to the laboratory to conduct immediate root excision, always during the first hours of the morning (before 9:00 a.m.) to prevent exposure to higher temperatures. Root tips were washed in distilled water and pre-treated in 2 MM 8-hydroxyquinoleine for 3 h, followed by a rinsing step in distilled water and then a fixation step in freshly prepared 3:1 96% ethanol–1 glacial acetic acid solution for 24 h. Samples were thoroughly rinsed in 70% ethanol to remove glacial acetic acid, since it has been described to interfere with later chromosome staining [58]. Samples were either stored in 70% ethanol at 4 °C for further use or directly used in the following steps. Root tips were rinsed in distilled water for 5 min, followed by an additional 5-min rinsing step in fresh distilled water. Samples were hydrolyzed by plunging the root tips in an Eppendorf tube with HCL 1 N for 6–9 min in a water bath at 60 °C. The tubes were pre-heated to bath temperature before root tip plunging. Two rinsing steps of 5 min each in distilled water followed to eliminate all possible HCL remains. For mitotic analysis, root tips were stained either in aceto-orcein for 48 h or in Schiff’s reagent for 12 h in dark conditions. Stained root apices were excised under a binocular magnifying glass, and immediately squashed on a slide. Cells were observed in a light optical microscope (an Olympus CH-40), and cells stained with Schiff’s reagent were also examined in a Leica TCS-SP2 confocal laser microscope (LEICA Microsystems Heidelberg GmbH, Mannheim, Germany) under a HCX PL APO CS 63.0 × 1.40 OIL objective, 580 nm–725 nm emission band, and excitation wavelength of 561 nm with a DPS (561 nm) laser diode. In several populations, when chromosome number was identified in one or a few individuals from a population, ploidy level confirmation was established by flow cytometry on silica-gel dried plants from the same population and/or from a close population. Flow cytometry measurements were conducted on a Cell Lab Quanta Beckman Coulter flow cytometer (Beckman Coulter, Chaska, MN, USA) with a 100 W mercury arc lamp and excitation line optimized at 366 nm for UV estimations of DNA content with DAPI fluorochrome. Dried samples conserved in silica gel were prepared by chopping in 600 µL of Galbraith’s buffer with 100 µg ml^−1^ RNaseA. *Pisum sativum* ‘Express Long’ (2C = 8.7 pg) was initially used as internal reference, although samples from known diploid level (2n = 12) of *Jasione montana* L. subsp. *montana* were used for rapid assessment of polyploid populations in other cases. Filtering was conducted in a 33 μm nylon filter, and 2 µg mL^−1^ of DAPI fluorochrome was added to the mixture. A total of 10,000 particles were measured in each sample, and the 2C- values were calculated. As dehydrated instead of fresh samples were used, precise genome size estimation was ruled out, with the objective being to grossly estimate the ploidy level of the sample by comparison of the position of plotted peaks.

### 4.4. Species Delimitation with ASAP

Assemble Species by Automatic Partitioning (ASAP) is a molecular method for species delimitation that uses group-specific ad hoc thresholds without any a priori species hypothesis, which are estimated by looking for the barcoding gap in the frequency distribution of nucleotide distances. It represents a transition between intraspecific and interspecific categories [32]. ASAP is based on a hierarchical clustering algorithm using only pairwise genetic distances from single locus sequence alignments to produce a species partition scheme ranked by a scored system [32]. The current authors used a set of plastid regions that are inherited together and, therefore, can be considered a single locus for species delimitation methods [59]. ASAP creates successive partitions from all terminals split to all those lumped into a single group and assigns them a probability of panmixia (*p*-value) that quantifies the partitions’ chances of being a single species. Then, it computes the width of the barcode gap between the previous state of partitions and after the new partitions are created. Probability and gap width are ranked and averaged into a single asap-score that is used to rank the groupings, the lower the better [32]. ASAP analyses were conducted in the program web interface (https://bioinfo.mnhn.fr/abi/public/asap (accessed on 14 December 2023)) with the three available substitution models, namely Jukes-Cantor (JC69) [60], Kimura 2P [61], and simple p-distances. The analyses were run firstly from a dataset with 14 sequences, including all detected haplotypes in the populations of the study group plus a sequence from a population of the hexaploid *Jasione crispa* subsp. *crispa* from the Pyrenees, and secondly from an expanded dataset including samples covering all geographic areas with populations of the studied groups, implying that some haplotypes were represented more than once in the nucleotide matrix.

### 4.5. Species Delimitation with GMYC

The Generalized Mixed Yule Coalescent (GMYC) model [62] was used to suggest hypothetical species as independent evolutionary units. GNMYC is an exploratory method using single-locus sequence datasets and an ultra-metric phylogenetic tree as inputs to estimate rates of branching to ascertain which parts of the phylogeny behave following a population (coalescent) model and which parts follow a speciation (Yule) model. The favored species partition maximizes the likelihood of the transition between branching coalescent rates and branching speciation rates in absolute time by using an ultra-metric tree, identifying a threshold between speciation and coalescent rates. This single threshold approach is based on the assumption that species are monophyletic, and, therefore, clades defined by a most recent common ancestor (MRCA) reflect diversification events and can be considered species, while branches descending from each of the MRCA nodes reflect coalescent events [59,62]. The set of nodes with the highest maximum likelihood is chosen as the best species partition model. There is a version of the methods that allows us to infer multiple thresholds, relaxing the assumption that all diversification events must occur before all coalescent events [63]. However, it has been demonstrated that this variant of the method is prone to over-splitting [59,64]; therefore, its use was discarded. From each group (putative species) sequences of at least six individuals have been included in the GMYC analysis. When more than one population was included in the sample, at least two individuals per population have been sequenced (mean 4.1 indiv./population). Fujisawa and Barraclough [59] observed that increases in the number of sampled individuals per species led to better performances with the method. The reason is that greater sampling might increase the detected branching rate and, therefore, the detection of a threshold in branching. However, greater species sampling, particularly without increasing within-species sampling of individuals, might reduce the ability of GMYC to detect species. The reason is that species monophyly is more easily inferred if random but incomplete sampling means that some close species are not included in the analysis [65]. Hence, with the aim of an earnest procedure and to avoid unrealistic over-split results, we have sampled the complete geographic range of the investigated taxa, even if it could hamper the detection of non-monophyletic species. As within-species geographic structure could affect the accuracy of the method, causing some relatively isolated populations to be delimited as separate species [66,67], the sampling design aimed to include not only the main geographic groups but all putatively linking areas. Nevertheless, simulations of the GMYC method showed that if the effective population size remains low relative to species divergence (that is, relatively low within-species variation) the threshold is optimized at the correct point of the tree and the effect of geographic structuring is minimal [59]. Moreover, Talavera et al. [68] observed that the removal of intermediate haplotypes had little effect in the results and did not delimit the most extreme haplotypes as different species. The analyses have been conducted by both including repeated sequences and pruning repeated sequences. GMYC performance is affected by identical sequences because terminal zero-length branches affect the likelihood estimation [59,63]. However, when the tree is produced using a genealogy-based inference, as is the case of BEAST, the program does not assign null lengths to identical terminals, since they are treated as different haplotypes coalescing to a MRCA node, which allows GMYC to handle them. Talavera et al. [68] did not observe differences in GMYC results when using repeated sequences from a tree inferred with BEAST relative to when they were removed, although they recommend collapsing all repeated sequences to only keep different haplotypes to reduce computational times. Conversely, Michonneau [10] advocated the use of repeated sequences in GMYC analysis using a tree inferred by BEAST. BEAST can overestimate effective population sizes if identical sequences are removed by inferring higher unrealistic levels of genetic diversity [10]. Since population sizes affect lineage coalescence, the removal of identical sequences would increase average branch length, hampering the distinction between divergent and coalescent events. Michonneau [10] considered that collapsing sequences to haplotypes would cause species over-splitting and higher uncertainty in the analysis. While the latter is a probably undesirable outcome, it could also be that keeping identical sequences from BEAST, with minimal (although not zero) lengths could push to the external nodes the species delimitation threshold. Thus, two datasets have been included, one with all identical sequences collapsed to distinct haplotypes (“haplotypes” dataset, the same as in ASAP) and other in which different sequences were retained, in a proportion somewhat similar at how they were found in natural populations across the geographic range of the taxa (the populations “dataset”). When one haplotype was found in only one population, it was represented only once in the dataset, whether it was the only haplotype in the population or not. The possibility that one species could be represented by only one sequence in the dataset (i.e., a singleton) has demonstrated no negative effects in the GMYC analysis [68].

GMYC analyses were carried out from trees estimated with BEAST2. The substitution model was HKY + G, with the gamma count categories prior set four categories, as a small number of categories of gamma-distributed rates of evolution is enough to capture rate variation in small dataset [69]. In the first dataset (collapsed to haplotypes) two BEAST priors were modified to test possible effects on the GMYC results. It is expected that variation in priors, such as branching pattern and rate of molecular evolution, would not affect the inferred topologies, but the branch lengths will vary. Two different priors were used for the expected branching, namely a Yule model, also called pure birth model [70], and a coalescent model with constant population size [71]. The former is expected when the tree has a constant speciation rate, while the latter describes branching patterns within the same species. For the Yule model analysis, the rate of molecular evolution was set as a “constant clock”, meaning that the amount of nucleotidic mutations increases at a constant rate with time, an assumption suitable for small datasets of very related taxa. We also tested a “relaxed clock”, meaning that mutation rates could vary throughout the tree following a log-normal distribution. For the coalescent model with constant population size, we only used a constant clock prior, as this is expected for within-species behaviour. The impact of prior variation has been studied in several works [10,63,68]. It has been indicated that the use of a coalescent constant population model would be more conservative than the Yule model because GMYC also uses a coalescent model as a null model of expected branching [63]. In fact, these authors estimated higher species number with the Yule model than with the constant coalescent model. However, since this parameter affects branch lengths, the prediction of how it would affect GMYC results is not straightforward and could be influenced by the amount of variation in the data [10].

We tested the effect of prior variation over GMYC results using the LRT test of significance. After that, for the GMYC analysis with the second dataset (with repeated sequences) it was inferred a Bayesian phylogeny with the coalescent constant population branching and constant clock molecular rate of the molecular evolution priors. Bayesian phylogenies were inferred in BEAST2, with 10 million generation MCMC lengths and 2 independent runs. Convergence was checked with Tracer v1.6 and, after inspection of the ESS values in each run, a 10% burn-in and posterior sample combination was carried out in LogCombiner. Finally, a maximum credibility clade tree was summarized with TreeAnnotator for each analysis. In the analysis with the relaxed clock model, prior parameters were left as default in BEAUti2, and the clock rate was set to 1.0.

BEAST ultrametric topologies and posterior probabilities were inspected in FigTree and exported in the Newick format, since it is required by the R package splits [72] which was used to apply the GMYC method with a single threshold. Each GMYC analysis was repeated in the GMYC server (https://species.h-its.org/gmyc (accessed on 14 December 2023)). Species delimitations, LRT significances, and GMYC (AIC) supports values were compared among the analyses.

### 4.6. Other Lines of Evidence: Morphology

The putative species delineated by the GMYC and ASAP methods can be interpreted as hypotheses and to determine whether there are other biologically sound lines of evidence that need to be investigated. Looking for delimitating morphological traits can be difficult if not misleading in a genus of which it has been said that “there is not any clear morphological discontinuity between two species of *Jasione*” [73]. Nevertheless, and with the guidance of GMYC and ASAP results, some subtle morphological characters are investigated here. The relative size of the epidermal cells of the adaxial and abaxial sides of the leaf has been pointed out by Ref. [34] as one of the few anatomical characters providing taxonomical information in *Jasione*. In most species studied by these authors, adaxial and abaxial epidermal cells were subequal in size. However, these author identified four Iberian *Jasione* taxa, including *J. sessiliflora* and *J. crispa* subsp. *tomentosa*, and the adaxial epidermal cells were 1.5–2 times larger than the abaxial epidermal cells. This trait was investigated here within the more homogeneous taxa of the model group, namely *Jasione sessiliflora* subsp. *appressifolia*, *Jasione crispa* subsp. *tomentosa*, *Jasione crispa* var. *praelittoralis*, and the less similar *Jasione crispa* subsp. *mariana*. Epidermal peelings of leaves from herbarium specimens were stained with blue toluidine and photographed at 20× magnification in a light microscope. The length of the cells’ largest dimension was measured in 25 adaxial and 25 abaxial epidermal cells in each leaf, with 10 leaves per individual. Ongoing phylogenetic analyses in the genus (*M. Serrano*, own data) showed that the occurrence of hydathodic structures on the upper side of the leaf have a distribution that correlates with the evolutionary history of the genus. Here, the average number of hydathodic structures per leaf was compared in the model group with the width of leaf hairs, a measure relative to the size and stiffness of leaf trichomes.

### 4.7. Biogeographical Regionalization a Species Delimitation

To test for the adequacy of the delimited entities to a pre-established biogeographical framework, the studied populations were superimposed onto a georeferenced map of the biogeographic subprovinces of the Iberian Peninsula (*M. Serrano*, own digitization), based on Ref. [17].

The haplotypes were organized in five main haplogroups, those with high posterior probability in the Bayesian genealogy, and excluding the Pyrenean *J. crispa* subsp. *crispa* hexaploid lineage. The frequency of the chloroplast haplogroups in the Iberian biogeographic subprovinces was surveyed and visualized using heatmaps. The heatmaps were generated in R v.4 with the ComplexHeatmaps package [74]. Two heatmap analyses were generated. First, a heatmap without internal clustering was produced in which Eurosiberian and Mediterranean subprovinces were ordered according to a thermic gradient. The gradient was based on the mean values of the climatic variable BIO11 (mean daily mean air temperatures of the coldest quarter) extracted from the Chelsa database [75], as an approach to cold ecological constraints during winter, for all the geographical presences in each subprovince of the studied groups. These occurrences are shown in Figure 7. Second, a heatmap with a split simultaneously performed by rows and columns, applying k-means partitioning analysis, was used to detect underlying patterns. The k parameter is set to three for rows to test whether the partitioning recovers the most probable delimited species number suggested by the GMYC and ASAP methods, that is “*praelittoralis*”, the remaining diploids, and the tetraploids. The k parameter is set to four for columns as it is the number of major inferred dendrogram clusters of subprovinces obtained by hierarchical clustering; therefore, we group them as Northwest, Southwest, Central, and East subprovinces. As the k-means analysis uses random start points, which could generate different clusters from different runs, the analysis was repeated 25 times to check the consistency of the results, observing no variation among them.

## 5. Conclusions

Species delineation tools based on co-inherited multilocus information from the chloroplast genome were useful to delineate undetected evolutionary significant entities (i.e., cryptic species) in a plant group with high levels of morphological stasis. Different complementary lines of evidence supported the main conclusion of the GMYC and ASAP species delimitation analyses. Analysis based on biogeographic regionalization, as a synthesis of environmental and historical information, also supported the suggested species delineation, contributing to making the species hypothesis biologically sound as an ecological unit.

## Figures and Tables

**Table 1:** ASAP analysis without repeated sequences. First two best-ranked ASAP partitions, indicating asap-scores for each substitution model, probability for panmixia (*p*-value), relative gap width metric (W), and inferred threshold distance among species. The results favor a 4 species partition, followed by a 6 species partition with similar asap-scores.

Species Number	Substitution Model	asap-Score	*p*-Value	W Rank	Threshold Dist.
4 *	JC69	2.0	7.23 × 10^−1^	4.69 × 10^−5^	0.000681
	K80	2.0	6.89 × 10^−1^		
	p-distances	2.5	6.07 × 10^−1^		
6	JC69	2.0	7.37 × 10^−1^	6.48 × 10^−5^	0.000454
	K80	2.0	7.09 × 10^−1^		
	p-distances	2.0	7.47 × 10^−1^		

* Indicates partitions above the reference range of distances.

**Table 2:** ASAP analysis with repeated sequences as they are found in different geographic areas. First two best-ranked ASAP partitions, indicating asap-scores for each substitution model, probability for panmixia (*p*-value), relative gap width metric (W), and inferred threshold distance among species. The results favor a 4 species partition, followed by a 6 species partition with similar asap-scores.

Species Number	Substitution Model	asap-Score	*p*-Value	W Rank	Threshold Dist.
4 *	JC69	2.00	5.45 × 10^−1^	6.21 × 10^−6^	0.000681
	K80	2.00	5.31 × 10^−1^		
	p-distances	2.00	5.37 × 10^−1^		
6	JC69	2.00	6.37 × 10^−1^	1.02 × 10^−5^	0.000454
	K80	2.00	6.87 × 10^−1^		
	p-distances	2.00	6.51 × 10^−1^		

* Indicates partitions above the reference range of distances.

**Table 3:** Summary of the GMYC maximum-likelihood estimates of species delimitation. n.s.: non-significant, ***: *p* ≤ 0.001.

Dataset Type	Haplotypes			Populations
Priors	Yule, constant clock	Yule, relaxed clock	Coalescent constant clock	Coalescent constant clock
Species number	4	2	4	4
LRT result	0.8815751 n.s.	0.9209454 n.s.	0.1572022 n.s.	0.0003451632 ***
ML entities, confidence interval	1–13	1–13	1–7	4–9
GMYC support (1)	0.44	0.24	0.60	0.86

GMYC support (AIC values) averaged from all nodes of the estimated putative species.

**Table 4:** List of primers used for each gene region.

DNA Region	Primer Name	Sequence	Reference
*ndh*F	5bF	5′ GGAGCTACTTTAGCTCTTG 3′	[55]
	10bR	5′ CCTACTCCATTTGGAATTCCATC 3′	
*trn*L-*trn*F	E	5′ GGTTCAAGTCCCTCTATCCC 3′	[56]
	F	5′ ATTTGAACTGGTGACACGAG 3′	
*trn*H-*psb*A	psbA-F	5′ GTTATGCATGAACGTAATGCTC 3′	[57]
	trnH-R	5′ CGCGCATGGTGGATTCACAAATC 3′	
*trn*T(UGU)-*trn*Q	trnQ-novo	5′ GCGTAGCCAAGYGGTAAGGC 3′	Designed here
	Jasi-trnQ-R	5′ TTTGGTCCCGGAAGTCGAAG 3′	

## Data Availability

The data generated in this work are available to those interested when requested.

## Supplementary Materials

The following supporting information can be downloaded at: https://www.mdpi.com/article/10.3390/plants12244176/s1, Table S1: Genbank accessions of plastid markers with information on ploidy, locality, and herbarium voucher. * Latitude and longitude precision in *Jasione crispa* var. *praelittoralis* is purposely obscured for conservation reasons.
