# Supplementary material for: Species Delimitation in a Polyploid Group of Iberian Jasione (Campanulaceae) Unveils Coherence between Cryptic Speciation and Biogeographical Regionalization
Source: Plants (Basel). 2023 Dec 15;12(24):4176. doi: 10.3390/plants12244176 (PMC10747609; doi:10.3390/plants12244176)
Supplement: Supplementary file 1 [file plants-12-04176-s001.zip › plants-2757053-supplementary.pdf]

Supplementary Materials

Table S1: Genbank accessions of plastid markers with information on ploidy, locality, and herbarium voucher. * Latitude and longitude precision in *Jasione crispa* var. *praelittoralis* is purposely obscured for conservation reasons.

Taxon	Ploidy level	Location and voucher	latitude	longitude	psbA-trnH	ndhF	trnL-trnF	trnT(UGU)-trnQ
J.crispa var. *praelittoralis*	2x	Spain, Castellfollit SANT62725	41.3*	1.0*	OR873368	OR873245	OR873286	OR873327
J. sessiliflora	4x	Spain, Laspaules SANT62894	42.472049	0.587369	OR873369	OR873246	OR873287	OR873328
J. sessiliflora	4x	Portugal, Devesas SANT70519	40.90534	-7.21958	OR873370	OR873247	OR873288	OR873329
J. sessiliflora	4x	Spain, Lobios SANT64249	41.833918	-8.077569	OR873371	OR873248	OR873289	OR873330
J. sessiliflora	4x	Spain, La Guiana SANT64376	42.442065	-6.602601	OR873372	OR873249	OR873290	OR873331
J. sessiliflora	4x	Spain, Ventanilla SANT60171	42.876833	-4.56027	OR873373	OR873250	OR873291	OR873332
J. sessiliflora	4x	Spain, Orihuela del Tremedal SANT64352	40.510161 3	-1.65522	OR873374	OR873251	OR873292	OR873333
J. sessiliflora	4x	Portugal, Formil SANT62915	41.798864	-6.842219	OR873375	OR873252	OR873293	OR873334
J. sessiliflora	4x	Portugal, Fundão SANT62467	40.11008	-7.475982	OR873376	OR873253	OR873294	OR873335

J. sessiliflora	4x	Spain, Valdemanco SANT62734	40.880619	-3.885966	OR873377	OR873254	OR873295	OR873336
J. sessiliflora	4x	Spain, Vinuesa SANT64298	41.862114	-2.733729	OR873378	OR873255	OR873296	OR873337
J. sessiliflora	4x	Spain, Candelario SANT76418	40.336592	-5.766513	OR873379	OR873256	OR873297	OR873338
J.crispa subsp. *segurensis*	4x	Spain, Orcera SANT62775	38.324479	-2.646675	OR873380	OR873257	OR873298	OR873339
J.crispa subsp. *segurensis*	4x	Spain, Riopar SANT83471	38.470780	-2.457637	OR873381	OR873258	OR873299	OR873340
"J. crispa"	4x	Spain, Gredos, SANT62778	40.269670	-5.239406	OR873382	OR873259	OR873300	OR873341
"J. crispa"	4x	Portugal, Serra da Estrela SANT62903	40.321060	-7.609320	OR873383	OR873260	OR873301	OR873342
J.crispa subsp. tomentosa	4x	Spain, Almagro SANT60478	38.856944	-3.663611	OR873384	OR873261	OR873302	OR873343
J.crispa subsp. tomentosa	4x	Spain, Aldea del Rey SANT60479	38.737222	-3.875833	OR873385	OR873262	OR873303	OR873344
J. sessiliflora subsp. *appressifolia*	2x	Spain, Torralba del Pinar SANT62765	39.974612	-0.429164	OR873386	OR873263	OR873304	OR873345
J. *sessiliflora* subsp. *appressifolia*	2x	Spain, Gàtova SANT62766	39.747875	-0.503215	OR873387	OR873264	OR873305	OR873346
J. sessiliflora subsp. *appressifolia*	2x	Spain, Alcudia de Veo SANT62765	39.901612	-0.376275	OR873388	OR873265	OR873306	OR873347
"J. crispa"	2x	Spain, Candelario SANT64366	40.333946	-5.729182	OR873389	OR873266	OR873307	OR873348
"J. crispa"	2x	Spain, Casares de las Hurdes SANT 70548	40.432053	-6.339712	OR873390	OR873267	OR873308	OR873349
J.crispa subsp. *tomentosa*	2x	Spain, Guadalupe SANT62675	39.470307	-5.40169	OR873391	OR873268	OR873309	OR873350
J.crispa subsp. *tomentosa*	2x	Spain, Hontanar SANT79493	39.585037	-4.526249	OR873392	OR873269	OR873310	OR873351

J.crispa subsp. *tomentosa*	2x	Spain, Puebla de Alcocer SANT62912	38.985098	-5.279662	OR873393	OR873270	OR873311	OR873352
J.crispa subsp. *tomentosa*	2x	Spain, Almodóvar del Campo SANT62892	38.597173	-4.78885	OR873394	OR873271	OR873312	OR873353
J.crispa subsp. *tomentosa*	2x	Spain, Poblete SANT77842	38.951100	-3.969200	OR873395	OR873272	OR873313	OR873354
J.crispa subsp. *tomentosa*	2x	Spain, Despeñaperros SANT64365	38.383046	-3.501806	OR873396	OR873273	OR873314	OR873355
J.crispa subsp. *mariana*	2x	Spain, Despeñaperros	38.383046	-3.501806	OR873397	OR873274	OR873315	OR873356
J.crispa subsp. *mariana*	2x	Spain, Santa Eufemia SANT72393	38.606894	-4.910859	OR873398	OR873275	OR873316	OR873357
J.crispa subsp. *mariana*	2x	Spain, Santa Eufemia SANT72394	38.606997	-4.910673	OR873399	OR873276	OR873317	OR873358
J.crispa subsp. *mariana*	2x	Spain, Villapalacios SANT79461	38.592707	-2.691179	OR873400	OR873277	OR873318	OR873359
J.crispa subsp. *tomentosa*	2x	Spain, Alhambra SANT62741	38.874781	-3.082159	OR873401	OR873278	OR873319	OR873360
J.crispa subsp. *tomentosa*	2x	Spain, Mora SANT64279	39.680148	-3.73019	OR873402	OR873279	OR873320	OR873361
J.crispa subsp. *tomentosa*	6x	Spain, Piedrabuena SANT62905	39.123514	-4.131192	OR873403	OR873280	OR873321	OR873362
J.crispa subsp. *crispa*	6x	Spain, Boí-Taüll SANT69663 ex1	42.479290	0.866214	OR873404	OR873281	OR873322	OR873363
J.crispa subsp. *crispa*	6x	Spain, Queralbs SANT60186	42.367510	2.152655	OR873405	OR873282	OR873323	OR873364
J.crispa subsp. *crispa*	6x	Andorra, Envalira SANT64353	42.538347	1.716657	OR873406	OR873283	OR873324	OR873365
J.crispa subsp. *crispa*	6x	Spain, Boí-Taüll 2 SANT69663 ex2	42.479290	0.866214	OR873407	OR873284	OR873325	OR873366
J.crispa subsp. *crispa*	6x	France, Pic d'Eina SANT83470	42.421563	2.153153	OR873408	OR873285	OR873326	OR873367
